# Brain-Machine Neurofeedback: Robotics or Electrical Stimulation?

**DOI:** 10.3389/fbioe.2020.00639

**Published:** 2020-07-07

**Authors:** Robert Guggenberger, Monika Heringhaus, Alireza Gharabaghi

**Affiliations:** Institute for Neuromodulation and Neurotechnology, Department of Neurosurgery and Neurotechnology, University of Tübingen, Tübingen, Germany

**Keywords:** neuromuscular electrical stimulation, brain-robot interface, brain-computer interface, state-dependent stimulation, closed-loop stimulation, robotic rehabilitation

## Abstract

Neurotechnology such as brain-machine interfaces (BMI) are currently being investigated as training devices for neurorehabilitation, when active movements are no longer possible. When the hand is paralyzed following a stroke for example, a robotic orthosis, functional electrical stimulation (FES) or their combination may provide movement assistance; i.e., the corresponding sensory and proprioceptive neurofeedback is given contingent to the movement intention or imagination, thereby closing the sensorimotor loop. Controlling these devices may be challenging or even frustrating. Direct comparisons between these two feedback modalities (robotics vs. FES) with regard to the workload they pose for the user are, however, missing. Twenty healthy subjects controlled a BMI by kinesthetic motor imagery of finger extension. Motor imagery-related sensorimotor desynchronization in the EEG beta frequency-band (17–21 Hz) was turned into passive opening of the contralateral hand by a robotic orthosis or FES in a randomized, cross-over block design. Mental demand, physical demand, temporal demand, performance, effort, and frustration level were captured with the NASA Task Load Index (NASA-TLX) questionnaire by comparing these workload components to each other (weights), evaluating them individually (ratings), and estimating the respective combinations (adjusted workload ratings). The findings were compared to the task-related aspects of active hand movement with EMG feedback. Furthermore, both feedback modalities were compared with regard to their BMI performance. Robotic and FES feedback had similar workloads when weighting and rating the different components. For both robotics and FES, mental demand was the most relevant component, and higher than during active movement with EMG feedback. The FES task led to significantly more physical (*p* = 0.0368) and less temporal demand (*p* = 0.0403) than the robotic task in the adjusted workload ratings. Notably, the FES task showed a physical demand 2.67 times closer to the EMG task, but a mental demand 6.79 times closer to the robotic task. On average, significantly more onsets were reached during the robotic as compared to the FES task (17.22 onsets, SD = 3.02 vs. 16.46, SD = 2.94 out of 20 opportunities; *p* = 0.016), even though there were no significant differences between the BMI classification accuracies of the conditions (*p* = 0.806; CI = −0.027 to −0.034). These findings may inform the design of neurorehabilitation interfaces toward human-centered hardware for a more natural bidirectional interaction and acceptance by the user.

## Introduction

About half of all severely affected stroke survivors remain with persistent motor deficits in the chronic disease stage despite therapeutic interventions on the basis of the current standard of care (Winters et al., 2015). Since these patients cannot use the affected hand for activities of daily living, novel interventions investigate different neurotechnological devices to facilitate upper limb motor rehabilitation, such as brain-machine interfaces (BMI), robotic orthoses, neuromuscular functional electrical stimulation (FES), and brain stimulation (Coscia et al., 2019). BMI approaches, for example, aim at closing the impaired sensorimotor loop in severe chronic stroke patients. They use robotic orthoses (Ang et al., 2015; Kasashima-Shindo et al., 2015; Belardinelli et al., 2017), FES devices (Kim et al., 2016; Biasiucci et al., 2018), and their combination (Grimm et al., 2016c; Resquín et al., 2017) to provide natural sensory and proprioceptive neurofeedback during movement intention or imagery. It is hypothesized that this approach will lead to reorganization of the corticospinal network through repetitive practice, and might ultimately restore the lost motor function (Naros and Gharabaghi, 2015, 2017; Belardinelli et al., 2017; Guggenberger et al., 2018).

However, these novel approaches often result in no relevant clinical improvements in severe chronic stroke patients yet (Coscia et al., 2019). Therefore, recent research has taken a refined and rather mechanistic approach, e.g., by targeting physiologically grounded and clinically relevant biomarkers with BMI neurofeedback; this has led to the conceptional differentiation between restorative therapeutic BMIs on the one side (as those applied in this study) and classical assistive BMIs on the other side like those applied to control devices such as wheel-chairs (Gharabaghi, 2016): While assistive BMIs intend to maximize the decoding accuracy, restorative BMIs want to enhance behaviorally relevant biomarkers. Specifically, brain oscillations in the beta frequency band have been suggested as potential candidate markers and therapeutic targets for technology-assisted stroke rehabilitation with restorative BMIs (Naros and Gharabaghi, 2015, 2017; Belardinelli et al., 2017), since they are known to enhance signal propagation in the motor system and to determine the input-output ratio of corticospinal excitability in a frequency- and phase-specific way (Raco et al., 2016; Khademi et al., 2018, 2019; Naros et al., 2019).

However, these restorative BMI devices differ from their predecessors, i.e., assistive BMIs, by an intentionally regularized and restricted feature space, e.g., by using the beta frequency band as a feedback signal for BMI control (Gharabaghi, 2016; Bauer and Gharabaghi, 2017). Such a more specific approach is inherently different from previous more flexible algorithms that select and weight brain signal features to maximize the decoding accuracy of the applied technology; restorative BMIs like the those applied in this study have, therefore, relevantly less classification accuracy than classical assistive BMIs (Vidaurre et al., 2011; Bryan et al., 2013). As the regularized and restricted feature space of such restorative BMI devices leads to a lower classification accuracy in comparison to more flexible approaches, it may be frustrating even for healthy participants (Fels et al., 2015). IN the context of the present study, we conjectured that such challenging tasks will increase the relevance of extraneous load aspects like the workload (Schnotz and Kürschner, 2007). Furthermore, the modulation range of the oscillatory beta frequency band is compromised in stroke patients, proportionally to their motor impairment level (Rossiter et al., 2014; Shiner et al., 2015). That means that more severely affected patients show less oscillatory event-related desynchronization (ERD) and synchronization (ERS) during motor execution or imagery (Pfurtscheller and Lopes da Silva, 1999). To our understanding, this underlines the relevance of beta oscillations as a therapeutic target for post-stroke rehabilitation. At the same time, however, this poses a major challenge for the affected patients and may, thereby, compromise their therapeutic benefit (Gomez-Rodriguez et al., 2011a,b; Brauchle et al., 2015).

To overcome these hurdles that are inherent to restorative BMI devices, we have investigated different approaches in the past: (i) Reducing the brain signal attenuation by the skull via the application of epidural interfaces (Gharabaghi et al., 2014b,c; Spüler et al., 2014), (ii) Augmenting the afferent feedback of the robotic orthosis by providing concurrent virtual reality input (Grimm et al., 2016a,b), (iii) combining the orthosis-assisted movements with neuromuscular (Grimm and Gharabaghi, 2016; Grimm et al., 2016c) or transcranial electrical stimulation (Naros et al., 2016a) to enhance the cortical modulation range (Reynolds et al., 2015), and (iv) optimizing the mental workload related to the use of BMI devices.

In this study, we focus on the latter approach, i.e., optimizing the mental workload related to the use of BMI devices. For the latter approach it would be necessary to better understand the workloads related to different technologies applied in the context of BMI feedback (robotics vs. FES). We, therefore, investigated the mental demand, physical demand, temporal demand, performance, effort, and frustration of healthy subjects controlling a BMI by motor imagery of finger extension. Motor imagery-related sensorimotor desynchronization in the beta frequency-band was turned into passive opening of the contralateral hand by a robotic exoskeleton or FES in a randomized, cross-over block design. The respective workloads were compared to the task-related aspects of active hand movement with EMG feedback. We conjectured a feedback-specific workload profile that would be informative for more personalized future BMI approaches.

## Methods

### Subjects

We recruited 20 healthy subjects (age = 23.5 ± 1.08 yeas [mean ± SD], range 19–27, 15 female) for this study. Subjects were not naive to the tasks. All were right-handed and reached a score equal or above 60 in the Edinburgh Handedness Inventory (Oldfield, 1971). The subjects gave their written informed consent before participation and the study protocol was approved by the Ethics Committee of the Medical Faculty of the University of Tübingen. They received monetary compensation.

### Subject Preparation

**Figure 1:**
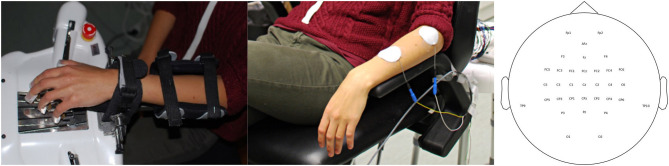
Experimental set-up. **(Left)** Robotic hand orthosis as feedback device (Amadeo, Tyromotion GmbH, Graz). **(Middle)** Neuromuscular forearm stimulation as feedback device (RehaMove 2, Hasomed GmbH, Magdeburg). In both cases, a brain-machine interface (BMI) detected motor imagery-related oscillations in the beta frequency band by an electroencephalogram (EEG) and provided via a BCI2000-system contingent feedback by moving the hand with either the robot or the electrical stimulation. **(Right)** The EEG montage used in this study.

We used Ag/AgCl electrodes in a 32 channel setup according to the international 10-20 system (Fp1, Fp2, F3, Fz, F4, FC5, FC3, FC1, FCz, FC2, FC4, FC6, C5, C3, C1, Cz, C2, C4, C6, TP9, CP5, CP3, CP1, CPz, CP2, CP4, CP6, P3, Pz, P4, O1, O2 with TP10 as Reference and AFz as Ground) to examine the cortical activation pattern during the training session. Electrode impedances were set below 10 kΩ. All signals are digitalized at a sampling frequency of 1,000 Hz (robotic block) or 5,000 Hz (FES block) using Brain Products Amplifiers and transmitted online to BCI2000 software. BCI2000 controlled in combination with a custom-made software the respective feedback device, i.e., either the robotic orthosis or the functional electrical stimulation. Depending on the task, one of the following preparations was performed. Either the robotic hand orthosis (Amadeo, Tyromotion) was attached to the subject's left hand (Figure 1A), fixated with Velcro strips across the forearm and with magnetic pads on the fingertips (Gharabaghi et al., 2014a; Naros et al., 2016b); or functional electrical stimulation (FES, Figure 1B) was applied to the M. extensor digitorum communis (EDC) by the RehaMove2 (Hasomed GmbH, Magdeburg) with two self-adhering electrodes (50 mm, HAN-SEN Trading & Consulting GmbH, Hamburg). First an electrode was fixed to the distal end of the EDC's muscle belly serving as ground. Then a rectangular electrode prepared with contact gel was used to find the optimal place for the second electrode where maximal extension of the left hand could be achieved. Here a custom written Matlab script was executed to detect the current threshold needed for the extension. Starting at 1 mA, the current was increased in steps of 0.5–1 mA. During each trial, FES was applied for 3 s with a pulse width of 1,000 μs and a frequency of 100 Hz. At the beginning of stimulation, a ramping protocol was implemented for 500 ms. Once, the correct position and threshold of stimulation were found, the temporary electrode was replaced by the second stimulation electrode and both were fixed with tape. A mean stimulation intensity of 6.5 mA (SD = 2.27) was required to cause the desired contraction in this study.

### Experimental Setup

**Figure 2:**
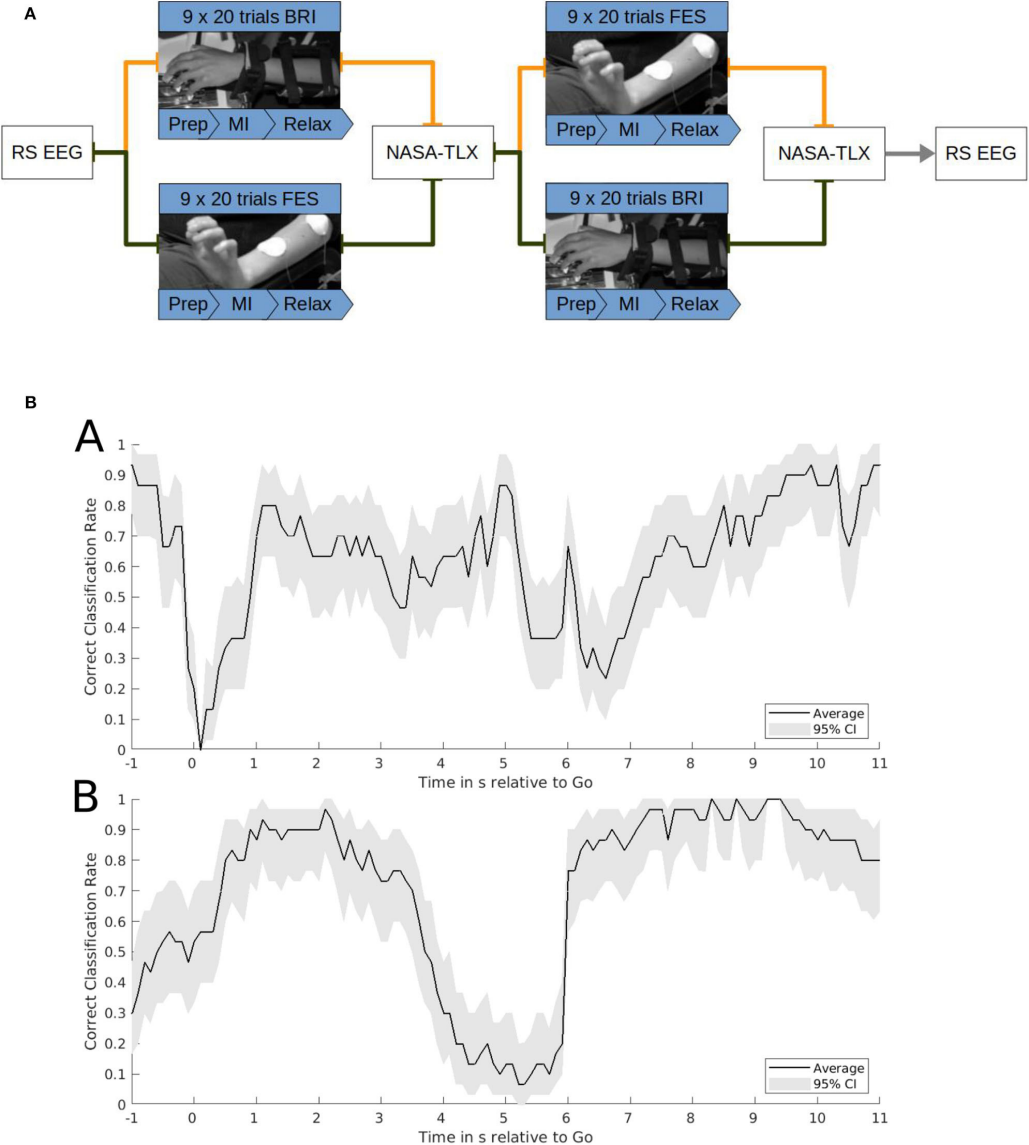
**(A)** Task structure. Functional electrical stimulation (FES) and robotic feedback were applied in a cross-over block design. The FES and robotic session consisted of 9 runs with 20 trials each. Each trial included a 2-s preparation phase, a 6-s motor imagery phase and a 6-s resting phase. One session lasted until at least 120 feedback onsets were reached. After each block subjects completed a NASA-TLX questionnaire. Before and after the intervention a resting state EEG was recorded. The study lasted ~3 h. **(B)** Correct classification rate (CCR). Exemplary single subject data of the CCR of brain-machine interface control with robotic feedback (a) or functional electrical stimulation (b). Time on the x-axis is relative to the go-cue for motor imagery at 0 s and the relax-cue at 6 s. The CCR on the y-axis indicates the probability of classifying the sample correctly as motor imagery after the go-cue or as rest after the relax-cue. It is calculated on the basis of a smoothened time course averaged over 30 trials with 95% confidence intervals estimated by bootstrapping.

In the beginning and end of the experiment, we recorded 3 min of resting state EEG measurements with the subjects having the eyes open. They were instructed to look straight ahead and focus on a white cross some 1.5 m in front of them on a screen. The study consisted of a motor imagery task with robotic feedback in one session and FES in the other. After each session the subjects completed a NASA Task Load Index (NASA-TLX) questionnaire (NASA Human Performance Research Group, 1986). The evaluation consisted of two parts. At first, the source of workload was identified: 15 cards were shown to the subject; each with two of the six categories mental demand, physical demand, temporal demand, performance, effort and frustration. The subject had to decide which of the respective two categories described the actual task demands better. Afterwards, scales from 0 to 100 were provided for all six categories, and the subjects were asked to rate each of them with regard to the respective task. The experimental structure is depicted in Figure 2A.

### Block and Trial Structure

Each session consisted of 9 runs of motor imagery-based feedback (either robotic or FES), with each run consisting of 20 trials. One session lasted until at least 120 feedback onsets (but no more than 139 onsets) were reached. A trial began with a preparation phase which was indicated by the auditory signal “left hand.” After 2 s, the subjects received a “go” cue. During the following 6 s, the hand robot or FES extended the fingers of the left hand if the classifier of the brain-machine interface (BMI) detected sufficient sensorimotor desynchronization in the contralateral sensorimotor cortex. Otherwise, the passive finger extension was stopped. Following this feedback period, the subject received an auditory cue to “relax,” after which the robot closed the subject's hand again. The relax period lasted 6 s, until the next trial commenced. Subjects were instructed to perform kinesthetic motor imagery of opening their left hands during the feedback period. They were instructed to keep calm and relaxed, and to refrain from performing any motor imagery during the relaxation period. For all trial periods, subjects were instructed not to perform any active movement.

### Classification Algorithm

As in our earlier studies, the classification algorithm was based on the average power in the beta-range (17–21 Hz) over sensorimotor electrodes (FC4, C4, and CP4), and has been described in detail before (Vukelić et al., 2014; Bauer et al., 2015, 2016b; Vukelić and Gharabaghi, 2015a): For online-analysis, we used BCI2000 (Schalk et al., 2004), implementing an autoregressive model based on the Burg Algorithm with a model order of 32 and a window size of 500 ms to update the beta-power value every 40 ms. The feature values were accumulated for the last 15 s of the resting period in a first-in-first-out manner. We calculated the standard deviation and mean of these buffered values to transform all estimates by subtraction of the mean and division with the standard-deviation into a z-scored distribution. Application of a threshold θ then enabled us to distinguish between sufficient and insufficient desynchronization relative to the average of the relaxation period. A classification of desynchronization as sufficient during the feedback period was considered a true-positive and caused extension of the fingers, while a classification as sufficient during the relaxation or preparation period was considered a false positive (Vukelić and Gharabaghi, 2015a,b; Bauer et al., 2016a,b). The desynchronization threshold that controlled the feedback was kept fixed at 0.6 for both the robotic movement and the FES.

### Offline Signal Processing

The data in the FES task was recorded with 5,000 Hz and then down-sampled to 1,000 Hz. Offline analysis was performed using an algorithm that was identical to the one described for online-analysis; therefore, no additional pre-processing was performed. For both tasks, the true positive rate (TP) and false positive rate (FP) were extracted from the three electrodes FC4, C4, and CP4, and the classification accuracy (CA) was calculated by the formula:

(1) $$\begin{array}{r} CA=\frac{TP+\left(1-FP\right)}{2} \end{array}$$

The first 2 s of the resting phase were excluded from analysis due to the closing of the robotic orthosis during this time period. Close attention was paid that there were no differences in the offline signal processing between the conditions and that equal time windows were used for the calculations.

This CA represents the mean performance of both the motor imagery and relax periods. To capture the instantaneous performance during each time-point of the tasks, the correct classification rate (CCR) needs to be estimated. The CCR indicates the true positive rate during the motor imagery period and the true negative rate during the rest period. Exemplary data for the CCR during the robotic and FES tasks is presented in Figure 2B.

### Statistical Analysis

One subject asked to quit the FES session after the first run, as the stimulation was painful even after repositioning of the electrodes and applying the lowest stimulation intensity. This subject was, therefore, excluded from the statistical analysis.

The analysis was divided into two steps.

First, the results of the NASA-TLX were investigated. The weights of each workload component were multiplied with the respective rating from 0 to 100. This resulted in the adjusted workload for each component. These adjusted workloads of the different categories were, finally, added up and divided by 6.

Afterwards, the means of total workloads, weights, ratings and adjusted workloads over all subjects were compared between the two feedback conditions. If the values were normally distributed on the basis of a Kolmogorov-Smirnov test, a bilateral *t*-test was applied; otherwise non-parametric methods, namely a sign test and a Wilcoxon sign-rank-test were used. All confidence intervals were calculated with a probability of 95%.

Furthermore, the weighted components of the NASA-TLX questionnaire of a previous study of our group by Fels et al. (2015) with actual hand opening and EMG feedback were included and compared to the two conditions of the present study in terms of likelihood relations between the workload weights.

Finally, the BMI performance of the two conditions (robotics vs. FES) was calculated and compared by estimating the CA, the TPR and the FPR, and recalculating them for thresholds between −5 and 5.

## Results

**Figure 3:**
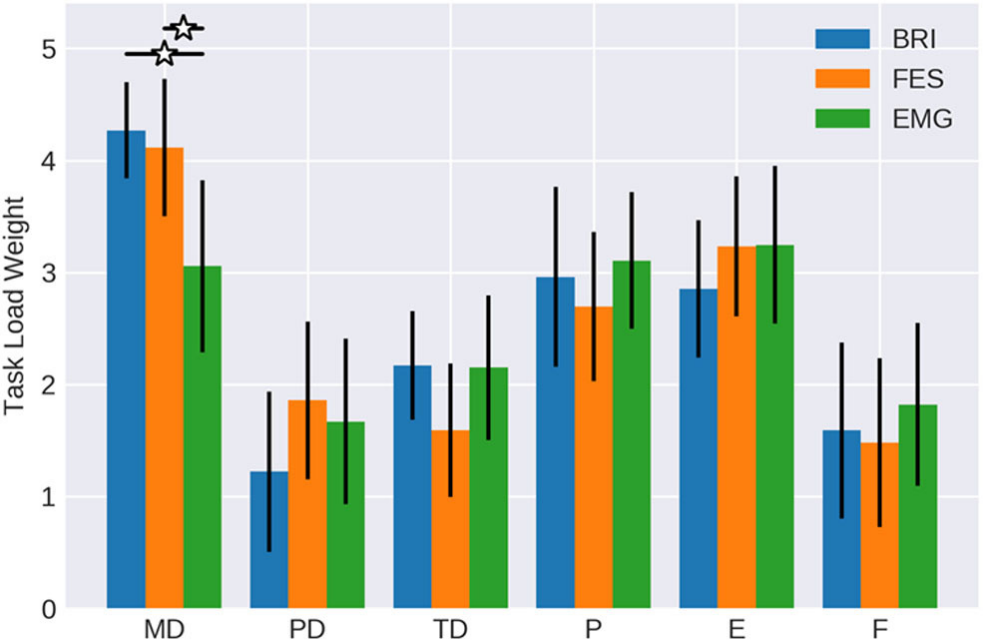
Weights of workload categories. The work load categories mental demand (MD), physical demand (PD), temporal demand (TD), performance (P), effort (E) and frustration (F) of the NASA-TLX for BMI control of passive movement with different feedback modalities (Robotic vs. FES) in comparison to active movement with EMG feedback (data form Fels et al., 2015). Confidence intervals are given with a probability of 95%.

The weights of the different workload components were quite similar between both robotic (brain-robot interfaces, BRI) and functional electrical stimulation (FES) feedback (see Table 1 and Figure 3). In both conditions, mental demand, performance and effort were considered as more relevant than frustration, physical demand, and temporal demand. Physical demand (*p* = 0.125) and temporal demand (*p* = 0.21) showed a trend to differ between conditions, whereas the other components did not (*p* > 0.79).

**Table 1:** Results for the task-load measures.

	**Weights**		**Magnitude**		**Score**	
	**BRI**	**FES**	**BRI**	**FES**	**BRI**	**FES**
Mental demand	4.26 ± 0.42	4.11 ± 0.59	76.32 ± 7.74	70.79 ± 8.08	328.9 ± 51.7	296.6 ± 57.5
Physical demand	1.21 ± 0.71	1.84 ± 0.71	27.63 ± 11.65	35.79 ± 12.08	58.7 ± 44.6	91.8 ± 48.9
Temporal demand	2.16 ± 0.49	1.58 ± 0.59	53.68 ± 11.04	44.84 ± 11.27	125.0 ± 40.9	78.0 ± 35.8
Performance	2.95 ± 0.79	2.68 ± 0.67	39.74 ± 11.6	41.58 ± 11.19	103.4 ± 42.0	98.7 ± 30.3
Effort	2.84 ± 0.61	3.21 ± 0.63	68.95 ± 9.76	67.90 ± 7.92	211.1 ± 57.1	225.3 ± 53.9
Frustration	1.58 ± 0.77	1.47 ± 0.75	42.90 ± 11.6	37.37 ± 11.65	96.6 ± 59.0	81.3 ± 50.9

*Numbers show in the columns the weights, the magnitude and the weighted score for the six different categories of the the NASA-TLX. For each task [i.e., brain-robot interface (BRI) vs. functional electrical stimulation (FES)], the table reports the mean and the width of the symmetric 95% confidence interval*.

When comparing the workload weights of the BMI tasks with the EMG task of the study of Fels et al. (2015), the mental demand of the latter was less (mean = 3.05; CI = 2.28 to 3.82) reaching levels similar to performance (mean = 3.1; CI = 2.49 to 3.70) and effort (mean = 3.24; CI = 2.55 to 3.93). The temporal demand (mean = 2.14; CI = 1.51 to 2.78), frustration (mean = 1.81; CI = 1.08 to 2.54), and physical demand (mean = 1.67; CI = 0.94 to 2.39) were similar between conditions.

Notably, it was 6.79 times more likely that the FES condition showed a mental demand like the robotic condition than the EMG task. Furthermore, the likelihood was at least 2.67 times greater for the FES than the robotic task to show a physical demand like the EMG task. All other components, showed no evident differences between conditions, i.e., values between 0.9 and 1.2.

**Figure 4:**
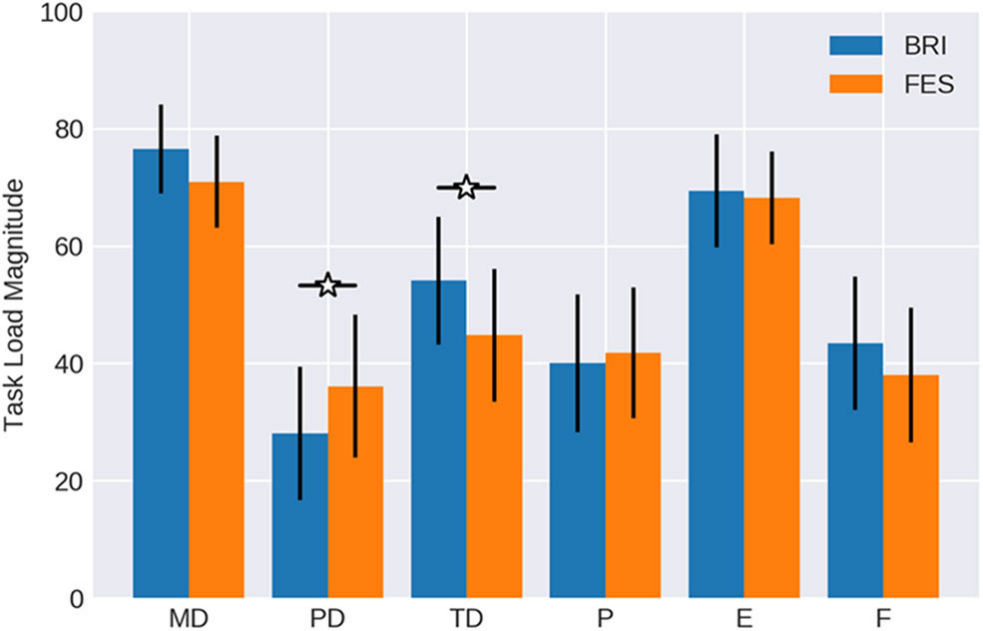
Magnitude of workload. Subjects rated the mental demand (MD), physical demand (PD), temporal demand (TD), performance (P), effort (E) and frustration (F) of the NASA-TLX for BMI control of passive movement with different feedback modalities (Robotic vs. FES) on a scale from 0 to 100. Data is averaged over all subjects. Confidence intervals are given with a probability of 95%.

The magnitude of workload for the different components (see Table 1 and Figure 4) was normally distributed with values greater than 0.24 in the Kolmogorov-Smirnov tests, and was similar to the weights. However, the physical demand of the FES task was significantly higher (*p* < 0.05) than of the robotic task. In contrast, the temporal demand was estimated significantly higher (*p* < 0.05) during the robotic task. On average, subjects stated that both tasks were mentally demanding and made the experience that they had to work hard to accomplish the performance level reflected in the effort value. Furthermore, most of them rated their performance as successful. Also, frustration was balanced in both conditions (*p* = 0.42).

**Figure 5:**
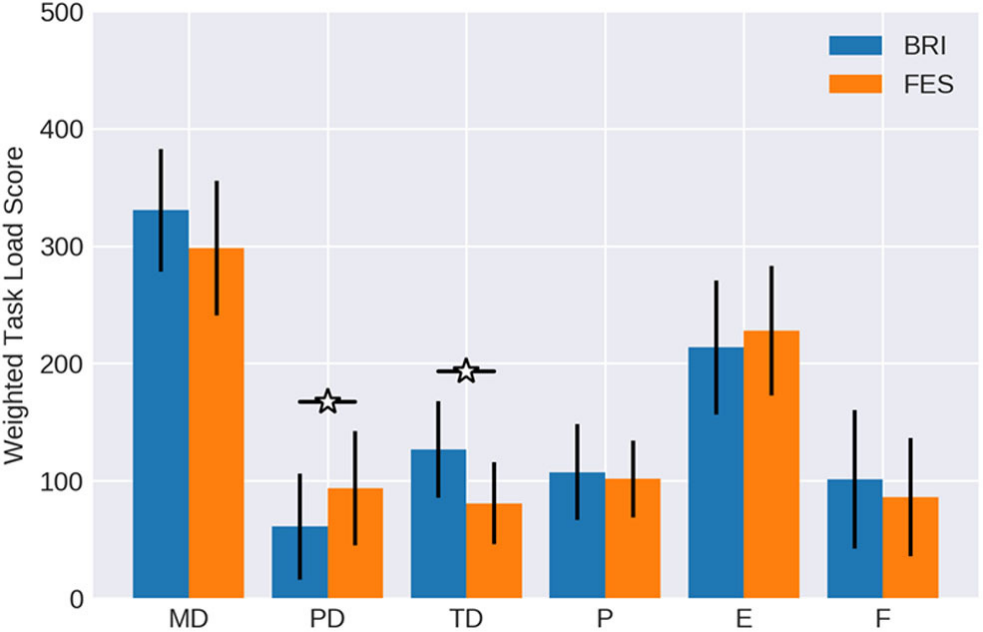
Weighted workload scores. The importance weight (Figure 3) of each workload component was multiplied with the respective magnitude (Figure 4) and averaged over all subjects. Confidence intervals are given with a probability of 95%.

Finally, two components were particularly relevant in the adjusted workload ratings (see Table 1 and Figure 5), which were normally distributed according to a Kolmogorov-Smirnov test with values greater than 0.23; namely mental demand and effort.

The remaining four components reached values only below 200. The differences between conditions with regard to the physical demand and temporal demand were significant in the adjusted ratings as well, similar to the findings for weight and rating. FES was estimated as significantly more physically demanding (*p* = 0.0368) than the robotic task. On the other hand, the temporal demand was indicated as significantly higher (*p* = 0.0403) when feedback was provided by the robotic hand orthosis. Performance and frustration did not show notable differences between conditions.

**Figure 6:**
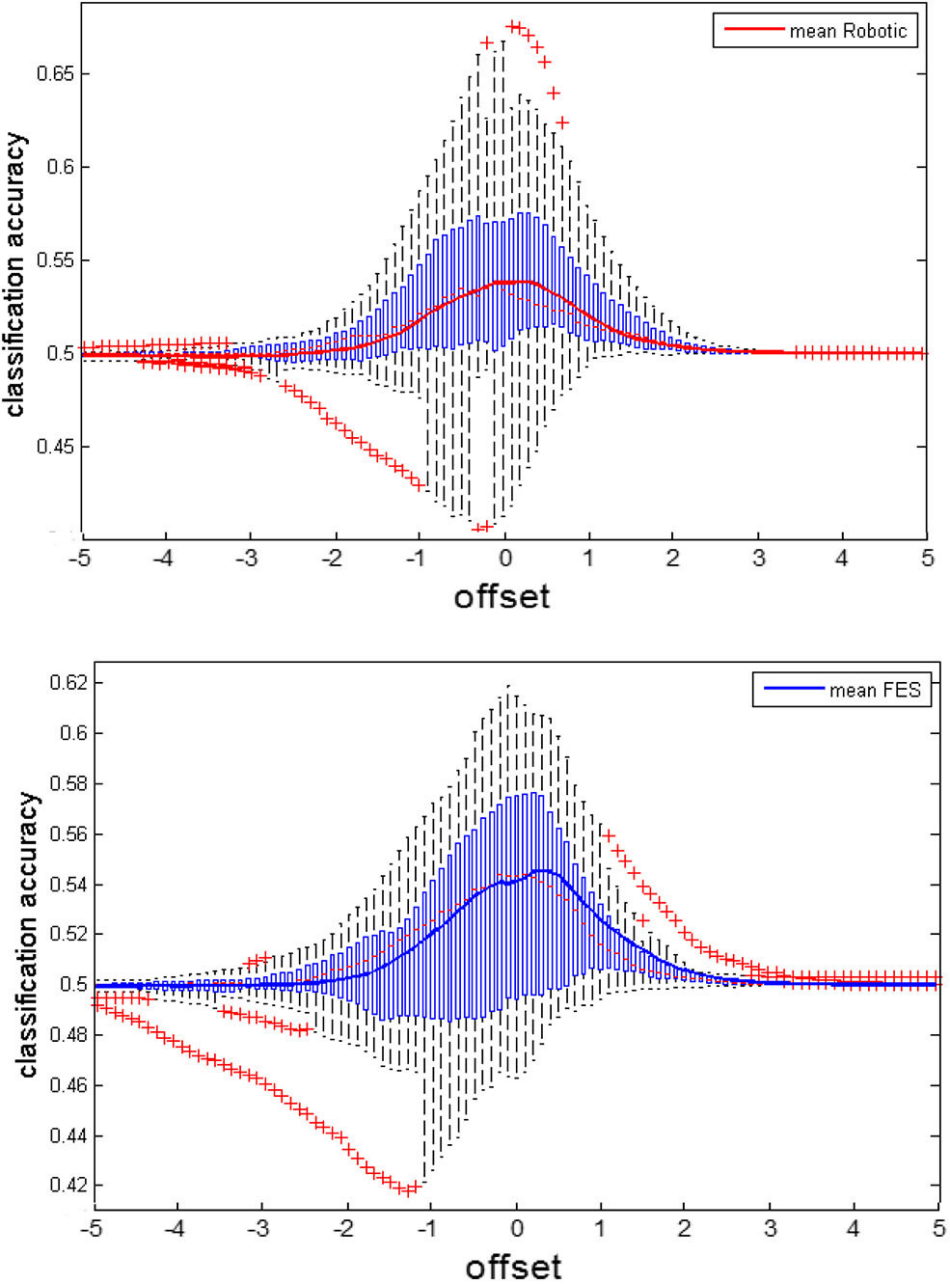
Classification accuracy of the feedback modalities. We recalculated the classification accuracy (CA) offline for various thresholds to estimate capacities for cognitive load (see also Bauer and Gharabaghi, 2015a). The subfigures show the average CA as solid trace, on top of boxplots indicating the distribution of CA within the group. Boxplots are characterized by the red dotted line indicating the median, the blue boxes indicating interquartile ranges (IQR) and the whiskers indicating 1.5 IQR. Red crosses mark extreme values outside the whiskers. The upper subplot shows the CA for the BRI task, the lower subplot for FES.

We calculated for each subjects the individual maximal classification accuracy at the optimal threshold. This maximal CA for BRI (61.579, CI = 54.05 to 69.11) and FES (mean = 58.112; CI = 50.37 to 65.85) was significantly above chance level, indicating that the subjects were able to control the respective orthosis. Notably, this maximal CA is different from the CA that is shown in Figure 6, which shows the CA averaged for each threshold. Specifically, a Gaussian fit of these classification accuracies across thresholds can be used to estimate the capacity for cognitive load while considering the instructional design of the task (see Bauer and Gharabaghi, 2015a). This analysis confirmed that there were no significant differences in this measure between conditions (*p* = 0.806; CI = −0.027 to −0.034); furthermore, i.e., neither the width (*p* = 0.553; CI = −0.280 to 0.155) nor the spatial location (*p* = 0.773; CI = −0.442 to 0.334) changed between conditions (Figure 6). However, while maximal CA did not differ, subjects could on average initiate orthosis movements in more trials with BRI (17.22/20, SD = 3.02) than with FES feedback (16.46/20, SD = 2.94). This difference was significant (*p* = 0.016). This indicates the following complementary findings: The performance in both tasks was different, i.e., subjects could to start the robotic orthosis slightly more often than the electrical one. However, the maximal attainable performance and cognitive load capacity was similar between the tasks.

## Discussion

This study showed that using a brain-machine interface with motor imagery and neurofeedback on the basis of a restricted and regularized feature space (i.e., sensorimotor beta frequency band) was a cognitively demanding task. It required relevant mental effort independent of the applied feedback modality even from young healthy subjects.

This observation may at least partially explain previous findings of limited added benefit, when applying robotics and FES during the neurorehabilitation of severely affected chronic stroke patients (McCabe et al., 2015). Specifically, when comparing the therapy outcome of standard physiotherapy on the basis of motor learning to that observed with additional robotics or FES, no differences were observed. The current mental demands of these neurotechnology-assisted interventions may be beyond the residual cognitive capacities of many stroke survivors, particularly of those with severe impairments. Along these lines, a recent meta-analysis revealed an association between cognitive deficits, particularly with regard to executive functions and attention, and arm motor recovery after stroke (Mullick et al., 2015).

**Figure 7:**
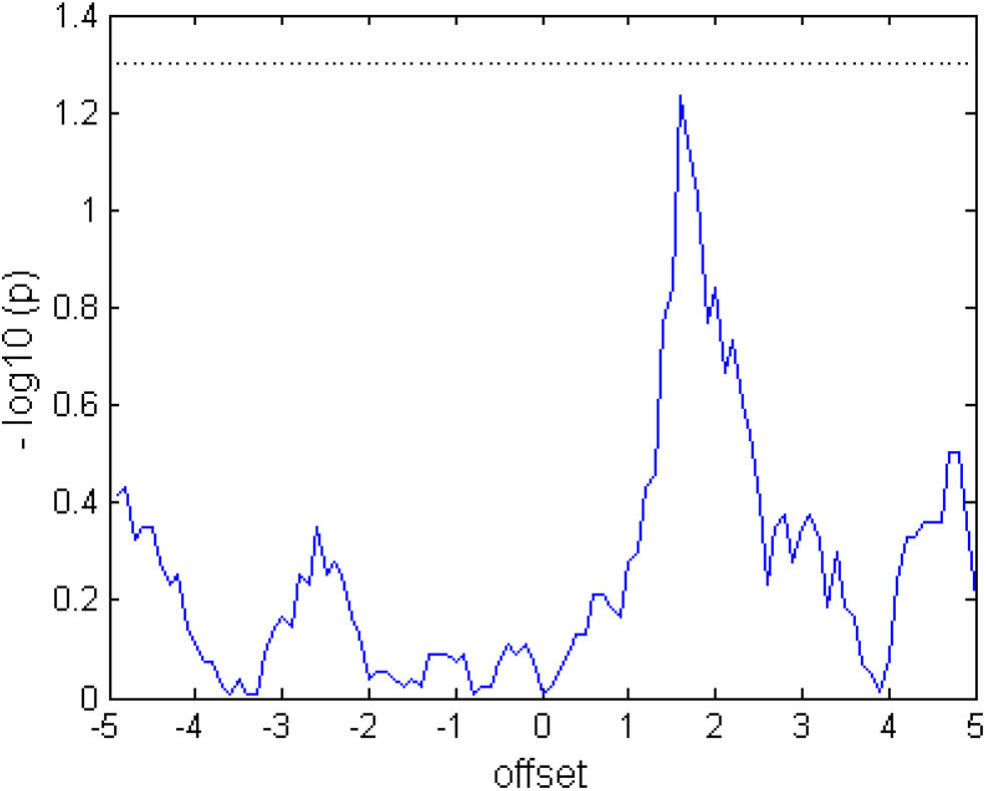
Comparing the classification accuracy (CA) between tasks. When the graph exceeds the dotted line at 1.3 (log10(p) after Wilcoxon signed rank test), the CA of the two tasks would differ at a significance level of *p* < 0.05. The values were calculated from the average over subjects and trials.

Presumably, due to the actual activation of muscles, physical demand was experienced significantly higher in FES that in robotics task, and thereby, more similar to the active movement condition with EMG feedback (Fels et al., 2015). This observation matches with studies showing muscular fatigue as a consequence of FES application (Doucet et al., 2012). We may only speculate about the significantly higher temporal demands of the robotics feedback. Since the device had to move back to the starting position in the resting phase of each trial, which took about 2 s, this may have been perceived as less intuitive that the instantaneous halt of the FES. In any case, these differences seem not to have impacted the BMI interaction during this single session intervention. Specifically, there were no differences in the classification accuracy between feedback conditions (see Figure 7) and only minor (even though significant) differences in the number of movement onsets (on average 17 robotic vs. 16 FES from 20 trials).

This indicates that the ability to perform the task remained unchanged during both conditions (Bauer and Gharabaghi, 2015a), and also that the opportunity to learn did not differ between the tasks (De Jong, 2010). This may be explained along the following lines. Even though robotics and FES led to rather “passive” and “active” movements, respectively, both were related to similar proprioception, i.e., perception of the current state of the limbs, which is mediated by muscle spindles (Naito et al., 2016). Therefore, passive movements have been shown to closely resemble actual motor execution with regard to the neuronal activation patterns (Szameitat et al., 2012; Bauer et al., 2015). This may have also caused the similar BMI performances with the two feedback modalities. However, the classification accuracies showed a large variability between subjects (Figure 6), a finding that suggests relevant differences between the participants with regard to the preference for one or the other feedback modality. This finding suggests, furthermore, that these preferences may be considered, when planning BMI interventions for patients.

### Future Research Directions

This work shows that the feedback modality (robotic vs. FES) seems not to be the major hurdle for translating BMI technology to effective clinical application, even though the role of different feedback modalities is still underexplored (Vukelić and Gharabaghi, 2015a; Kraus et al., 2016b, 2018; Royter and Gharabaghi, 2016; Darvishi et al., 2017). To overcome the inherent cognitive demands of this restorative neurotechnology, without sub-challenging the participants, physiologically-informed, online adaptations of task difficulty in the course of the training may be necessary.

It has previously been shown that cognitively demanding BMI tasks activated a distributed oscillatory network beyond the sensorimotor cortex that was trained by the neurofeedback (Vukelić et al., 2014; Bauer et al., 2015; Vukelić and Gharabaghi, 2015a,b). Addressing more specifically distinct parts of this network that mediate corticospinal gain modulation (Khademi et al., 2018) and subserve motor or cognitive control (Wagner et al., 2016) may overcome some of the current limitations. Importantly, future studies will need to evaluate in particular the learning process in subsequent training sessions (Naros et al., 2016b).

Moreover, the large variability of classification accuracies that we observed in this study indicates the need to detect the individually optimal task difficulty for each BMI user. This has already inspired approaches to adapt the classification threshold in the course of an intervention to overcome cognitive load issues, maintain motivation and improve reinforcement learning (Bauer and Gharabaghi, 2015a,b; Bauer et al., 2016a,b). However, classical BMI metrics like the classification accuracy may be suboptimal for such adaptations. A Bayesian simulation, for example, indicated that the difficulty threshold with the highest classification accuracy allowed for fast initial learning, but was suboptimal for retention (Bauer and Gharabaghi, 2015b). Accordingly, the optimal difficulty threshold from a motor learning perspective was defined as the result of an interaction with the individual's ability (Guadagnoli and Lee, 2004) and needed, therefore, to be sufficiently challenging, i.e., allowing for a fixed failure (Wilson et al., 2019).

Along these lines, we have previously shown that adaptation on the basis of self-rated mental effort improved the performance of BMI neurofeedback on the basis of beta oscillations (Bauer et al., 2016a). In this work, a linear relationship between the difficulty threshold and the self-rated mental effort was observed, and the threshold for optimal effort was significantly higher than the threshold for optimal classification accuracy. This, in turn, indicates that neurofeedback training at difficulty thresholds with higher mental efforts may improve learning, and that the mental demands related to BMI training may in fact be even beneficial if the participants are not overstrained. In this context, an online-adaptation strategy based on biomarkers of cognitive demand may be particularly important (Bauer et al., 2016a).

### Limitations

The single session, cross-over design of our study revealed instantaneous work load profiles related to BMI neurofeedback with different feedback modalities, but did not permit us to investigate the cumulative effects during subsequent interventions, which will have a relevant impact on cognitive load, motivation and learning. Beyond BMI performance metrics we did not evaluate other behavioral or physiological parameters, which may have helped to further differentiate between the feedback modalities. Cortical motor mapping with refined transcranial magnetic stimulation protocols (Kraus and Gharabaghi, 2015, 2016; Mathew et al., 2016), for example, may overcome this limitation and provide further insight into the differential modulation of sensorimotor areas by these neurotechnologies (Kraus et al., 2016a). The findings were, furthermore, acquired in healthy young participants, who may relevantly differ in their cognitive capacities and neurophysiological status (Mary et al., 2015) from the target population of these interventions. Future studies will therefore need to investigate BMI-related workload profiles in stroke patients and age-matched controls, and consider gender differences (Catrambone et al., 2019).

## Conclusion

Brain-machine interfaces are cognitively demanding independent of the applied feedback technology. Work load profiles help to design more personalized neurorehabilitation interfaces tailored to the individual needs of BMI users. This may facilitate human-centered rehabilitation hardware for a more natural bidirectional interaction and acceptance by the user.

## Data Availability Statement

The raw data supporting the conclusions of this article will be made available by the authors, without undue reservation, to any qualified researcher.

## Ethics Statement

The studies involving human participants were reviewed and approved by Ethics Committee of the Medical Faculty of the University of Tübingen. The participants provided their written informed consent prior to participation in this study.

## Author Contributions

RG and MH: conceptualization, methodology, formal analysis, writing—review, and editing. AG: conceptualization, supervision, project administration, fund acquisition, and writing—original draft. All authors contributed to the article and approved the submitted version.

## Conflict of Interest

The authors declare that the research was conducted in the absence of any commercial or financial relationships that could be construed as a potential conflict of interest.
